# Tuning the Optical Properties of Hyperbolic Metamaterials by Controlling the Volume Fraction of Metallic Nanorods

**DOI:** 10.3390/nano9050739

**Published:** 2019-05-14

**Authors:** Alexey P. Leontiev, Olga Yu. Volkova, Irina A. Kolmychek, Anastasia V. Venets, Alexander R. Pomozov, Vasily S. Stolyarov, Tatiana V. Murzina, Kirill S. Napolskii

**Affiliations:** 1 Department of Materials Science, Lomonosov Moscow State University, 119991 Moscow, Russia; leontyevalecsey@mail.ru (A.P.L.); mv253@yandex.ru (O.Y.V.); 2 Department of Physics, Lomonosov Moscow State University, 119991 Moscow, Russia; irisha@shg.ru (I.A.K.); venets.n@mail.ru (A.V.V.); ar.pomozov@physics.msu.ru (A.R.P.); mur@shg.ru (T.V.M.); 3 Moscow Institute of Physics and Technology, 141700 Dolgoprudny, Russia; vasiliy.stoliarov@gmail.com; 4 Institute of Solid State Physics RAS, 142432 Chernogolovka, Russia; 5 All-Russian Research Institute of Automatics n.a. N.L. Dukhov (VNIIA), 127055, 119991 Moscow, Russia; 6 Department of Chemistry, Lomonosov Moscow State University, 119991 Moscow, Russia

**Keywords:** nanocomposite, templated electrodeposition, anodic aluminum oxide, array of nanorods, gold electrodeposition, hyperbolic metamaterial

## Abstract

Porous films of anodic aluminum oxide are widely used as templates for the electrochemical preparation of functional nanocomposites containing ordered arrays of anisotropic nanostructures. In these structures, the volume fraction of the inclusion phase, which strongly determines the functional properties of the nanocomposite, is equal to the porosity of the initial template. For the range of systems, the most pronounced effects and the best functional properties are expected when the volume fraction of metal is less than 10%, whereas the porosity of anodic aluminum oxide typically exceeds this value. In the present work, the possibility of the application of anodic aluminum oxide for obtaining hyperbolic metamaterials in the form of nanocomposites with the metal volume fraction smaller than the template porosity is demonstrated for the first time. A decrease in the fraction of the pores accessible for electrodeposition is achieved by controlled blocking of the portion of pores during anodization when the template is formed. The effectiveness of the proposed approach has been shown in the example of obtaining nanocomposites containing Au nanorods arrays. The possibility for the control over the position of the resonance absorption band corresponding to the excitation of collective longitudinal oscillations of the electron gas in the nanorods in a wide range of wavelengths by controlled decreasing of the metal volume fraction, is shown.

## 1. Introduction

Nanocomposites, owing to the synergy of matrix and inclusion phase properties, have found wide applications in many areas of science and technology [1,2,3]. Physicochemical properties of nanocomposites, including optical [4,5], mechanical [2], and catalytic ones [3], can be tuned by controlling the volume fraction of the inclusion phase. As an actual example of nanocomposites, ordered arrays of metallic nanorods in a dielectric matrix [6] actively studied as hyperbolic metamaterials [6,7,8,9,10] are worth being considered. Due to the strong structural anisotropy, the resonance effects in such materials appear in the two critical points corresponding to a zero value (epsilon near zero, ENZ) and a pole of the dielectric constant (epsilon near pole, ENP). The spectral position of these points and, as a consequence, optical properties of the metamaterial can be controlled by changing the chemical composition of the components of a nanocomposite or by variation of their geometric parameters [11,12,13]. For instance, the increase in metal volume fraction leads to shifting of the ENZ point to shorter wavelengths [14].

To obtain hyperbolic metamaterials based on an array of metallic nanorods in a dielectric matrix as a template, porous films of anodic aluminum oxide (AAO) containing an array of highly ordered pores and possessing high transmission in the visible spectral range are frequently used [11]. When the AAO template is completely filled with metal, the volume fraction of the metal coincides with the AAO porosity [15]. The metal fraction can be increased by preliminary chemical pore widening [14]. However, the best functional properties (such as high transparency and the possibility to control the ENZ resonance band position) are expected when the volume fraction of metal is smaller than 10%, whereas the porosity of anodic aluminum oxide exceeds this value. To reduce the porosity of AAO templates, the post-treatment of the films after anodization using the atomic layer deposition technique can be applied [16]. Although this method allows one to cover the pore walls by the layer of desired material with uniform thickness, it is an extremely time- and resource-consuming process. It is worth noting the chemical approach to partial filling of the pores by polymer before electrodeposition and, thus to decrease the volume fraction of the pores accessible to the electrodeposition [17,18]. In this case, the polymer fills the center of the pore, whereas the electrodeposition of metal leads to nanotubes formation.

In this work, the simple method for obtaining the hyperbolic metamaterials in the form of nanocomposites based on anodic aluminum oxide with the metal volume fraction smaller than the template porosity is proposed. The approach rests on the fact that during the increase in anodization voltage when the AAO film is being formed, a certain portion of pores stops growing and cannot be filled with metal in the subsequent course of electrodeposition [19,20]. For the quantitative analysis of the fraction of the pores filled with metal, scanning electron microscopy (SEM) images of the surface of the nanocomposites obtained after the selective removal of a current collector were statistically analyzed. The deviation of the experimentally determined value from the theoretical one allowed us to estimate the quantity of terminated, branched, and straight pores in the structure of the AAO films formed during the potentiostatic anodization of aluminum. The efficiency of the developed technique is demonstrated in the example of the controlled variation of the optical properties of the hyperbolic metamaterials based on the arrays of Au nanorods in the anodic aluminum matrix.

## 2. Materials and Methods

High-purity aluminum foils (100 µm thick, 99.99%) were used as a starting material. Prior to anodization, electrochemical polishing of aluminum in a solution containing 13 M H_3_PO_4_ and 1.85 M CrO_3_ at 80 °C in a pulse mode was performed as described elsewhere [21]. Forty pulses with a duration of 3 s and pulse-to-pulse interval of 40 s were imposed at an anodic current density of 0.4 A·cm^−2^. Then, aluminum was washed with deionized water and finally dried in air at room temperature.

Aluminum two-stage anodization was carried out in a two-electrode electrochemical cell in 0.3 M oxalic acid electrolyte using the DC power supply. The aluminum foil was pressed to the bottom side of the cell with an O-ring seal. The electroactive surface area of the anode was 8.0 cm^2^, whereas the aluminum ring of several times higher surface area was used as a cathode. The distance between the electrodes was equal to 10 cm. During anodization the electrolyte was vigorously agitated using an overhead stirrer and its temperature was kept constant at 20 °C using a thermostat.

**Figure 1:**
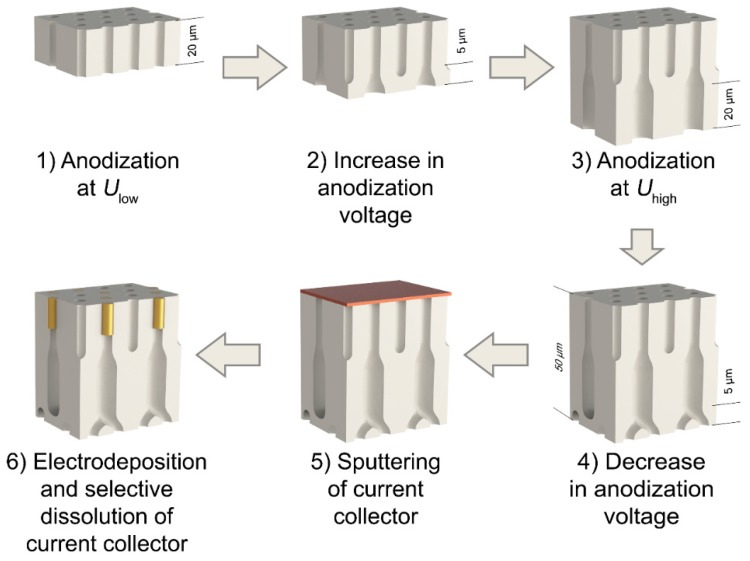
The scheme of the preparation of metal/anodic alumina nanocomposites with controlled fraction of the filled pores.

**Figure 2:**
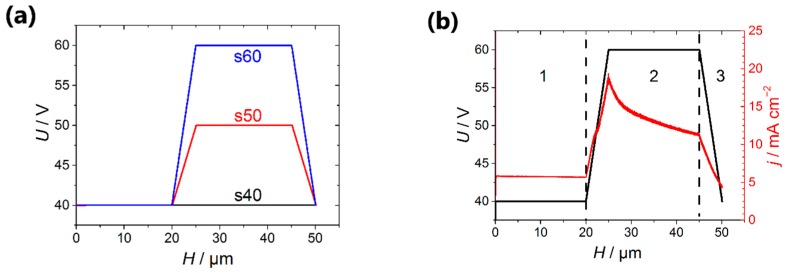
(**a**) The programs of aluminum anodization in 0.3 V oxalic acid at the temperature of 20 °C, which was used for the obtaining of porous films with partially blocked channels; (**b**) the dependences of the applied voltage (black line) and registered current density (red line) on the thickness of the porous film recorded at the second anodization stage in the case of the use of the template s60. The regions 1, 2 and 3 correspond to the following processes: 1) The formation of the upper part of the template designed for the electrodeposition of metal; 2) the blocking of a certain pores fraction; 3) the thinning of the barrier layer. The thickness of the porous film formed during the first anodization was 10 μm.

The first anodization stage was carried out at the voltage of 40 V until the charge corresponding to the AAO thickness of 10 μm was reached. The thickness of alumina films was controlled coulometrically using the experimentally found thickness-to-charge density ratio of 481 ± 9 nm·cm^2^·C^−1^. Then, the formed sacrificial layer of AAO was selectively etched away in a solution containing 0.5 M H_3_PO_4_ and 0.2 M CrO_3_ at 70 °C. The second anodization stage was carried out using the recently proposed voltage compared to the charge density regime [22] according to the following algorithm:

1) Twenty-μm thick AAO layer was prepared at the constant voltage of 40 V (step 1 in Figure 1).
2) The voltage was increased from 40 V to the high value (*U*_high_ = 50 V for the template denoted as s50 and *U*_high_ = 60 V in the case of the template s60) linearly with a charge passed during anodization (step 2 in Figure 1). If the templates were formed at a constant voltage of 40 V, they were denoted as s40 (Figure 2a). To produce transitional layers with the same thickness of 5 µm for the samples with a various value of the high voltage, the rate of voltage increase of 0.96 and 1.92 V·cm^2^·C^−1^ was used for the preparation of templates s50 and s60, respectively.
3) Potentiostatic anodization at the high voltage was carried out until the entire porous film thickness reached 45 μm (step 3 in Figure 1).
4) Anodization voltage was reduced to an initial value of 40 V linearly with a charge passed during anodization with the rate of 0.96 and 1.92 V·cm^2^·C^−1^ for the samples s50 and s60, respectively. Thus, the whole thickness of the AAO templates after this stage was 50 μm (step 4 in Figure 1).

The remaining aluminum was selectively dissolved in a 4 M solution of bromine in methanol. Then, the barrier oxide layer was removed by chemical etching in a 3 M H_3_PO_4_ solution at room temperature with the electrochemical detection of the pore opening moment. After this, a 200 nm thick copper layer was deposited onto the upper side (opposite to the side where the barrier layer was) of the through-hole AAO film (step 5 in Figure 1) by RF magnetron sputtering.

To prepare metal/anodic aluminum oxide nanocomposites, the templated electrodeposition of gold into AAO porous films was carried out. Electrodeposition was conducted at room temperature in a three-electrode electrochemical cell from a commercial electrolyte Ecomet 04-ZG containing buffered 50 mM [Au(CN)_2_]^−^ (pH 6). The platinum wire ring with a diameter of 2 cm served as a counter electrode. Saturated (KCl) Ag/AgCl electrode connected with the cell through a Luggin capillary was used as a reference electrode. Electrodeposition was performed in a potentiostatic mode at the deposition potential of −1 V. The deposition pulse of −1.4 V during 0.1 s was applied to provide instantaneous nucleation of metal particles. Here and below, all potentials are given in the scale of an Ag/AgCl reference electrode. During electrodeposition the electrolyte was agitated by a magnetic stirrer. To obtain nanocomposites with an equal length of the nanorods, the deposition charge was decreased proportionally to the number of the pores accessible for electrodeposition. Upon completion of the electrodeposition process, the copper current collector was selectively dissolved in a mixture containing 0.5 M H_2_O_2_ and 1 M H_2_SO_4_ solution under sonication (step 6 in Figure 1). Then, the sample was washed in deionized water and dried in airflow at room temperature.

The obtained samples of nanocomposites are denoted as ‘sXX_YYY nm’, where XX is the value of the high voltage (*U*_high_) applied during fabrication of a template and YYY is the average length of nanorods in nanometers.

Morphology of the samples was characterized using scanning electron microscopy. SEM images were collected using the scanning electron microscope NVision 40 (Carl Zeiss). Prior to SEM investigations, a 5 nm thick layer of chromium was deposited onto the surface of the samples by magnetron sputtering. Statistical analysis of SEM images was performed using the lab-made software [23]. The transmittance of the nanocomposites was studied using a halogen lamp as a broadband p-polarized light source for a wide range of the incidence angles, the transmitted light spectrum was detected with a spectrometer.

## 3. Results and Discussion

During the templated electrodeposition of metal in AAO, the formation of nuclei occurs on the bases of all the pores through which the access of the electrolyte to the electrode surface is provided. Then, under optimized electrodeposition conditions, it becomes possible to obtain an array of uniform in length nanowires with the controlled aspect ratio [24]. In this case, the volume fraction of metal is close to the template porosity. Hereinafter, for the calculation of the metal volume fraction, its volume is normalized to the volume of the filled portion of the template but not to the entire volume of the porous film. In the case of diffusion-controlled electrodeposition, the uniformity of the nanowires growth front can be disturbed, and at a high overvoltage and high rate of hydrogen release, the growth of metal in some pores can be stopped due to the pores being blocked by the gas bubbles. It is obvious that the increase in overvoltage cannot be considered as the method to diminish the metal volume fraction as it does not allow to preserve the planarity of the growth front.

An alternative way to obtain nanocomposites based on anodic aluminum oxide with the metal volume fraction smaller than the template porosity is the blocking of the certain portion of the channels in the oxide film. In the present work, it was achieved by the elevation of the anodization voltage after the formation of the main part of the template required for the metal deposition. When the anodization voltage is increased by $\sqrt{n}$ times, only 1/*n* of the channels continue to grow and other pores stop their growth [19,25,26] because the average interpore distance enlarges with the increase in the anodization voltage. When the current collector is deposited on the upper side of the porous AAO film with partially blocked channels, only pores accessible to the electrolyte are filled with metal during the subsequent electrocrystallization (Figure 1) [27]. Thus, the volume fraction of metal does not exceed *p*/*n* (where *p* is the porosity of the template).

The diameter distribution of the nanorods is determined by the geometric parameters of the porous structure. To obtain an ordered array of cylindrical channels with narrow size distribution in the upper part of the matrix, the two-stage anodization technique was applied [28]. At the first stage of anodization in 0.3 M oxalic acid at the voltage of 40 V and the temperature of 20 °C, the 10 μm-thick AAO layer was obtained. At such a thickness, even at an elevated temperature, the AAO film forms in the kinetic regime, thus allowing the acceleration of anodization without disturbing the process of self-ordering of the porous structure [29].

After the selective removal of the AAO sacrificial layer, the aluminum was re-anodized according to the procedure described in the Materials and Methods part. Two series of AAO templates were obtained in which the anodization voltage at the pores blocking stage achieved 50 V (template s50) and 60 V (template s60), respectively (Figure 2a). Porous 50-μm-thick AAO film obtained at the constant voltage of 40 V was used as a reference sample (template s40).

To obtain hyperbolic metamaterials, gold was deposited into the AAO template. The rapid and uniform growth of the nanorods was observed at the deposition potential of −1 V, the metal electrodeposition time being less than 11 min.

**Figure 3:**
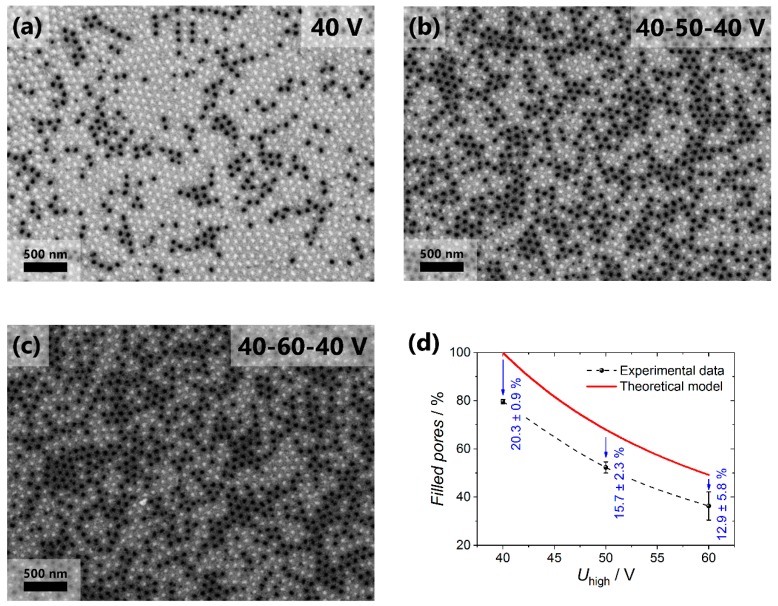
SEM images of the Au/AAO nanocomposites surface after selective dissolution of copper current collector: (**a**) Sample s40 without the blocking layer; (**b**) sample s50 with the blocking layer formed at 50 V; (**c**) sample s60 with the blocking layer formed at 60 V. Panel (**d**) demonstrates the comparison of the fraction of the filled pores with predictions according to Equation 1 as a function of the anodization voltage applied during the blocking layer formation.

Figure 3a–c shows SEM images of the surface of the nanocomposites after the selective removal of the copper current collector in the acidified solution of hydrogen peroxide. The light-colored pores on the images correspond to the channels filled with gold, and the black-colored pores are empty. The diameter of nanorods is 52 ± 5 nm for all the samples. With the increase in *U*_high_, the fraction of the filled channels decreases, which is caused by the blocking of a certain portion of pores (Figure 3d). The fraction of the pores accessible to the electrodeposition was calculated using the interpore distance by the formula:

(1) $$\varphi ={\left(\frac{D_{\mathrm{int}}^{high}}{D_{\mathrm{int}}^{low}}\right)}^{2}\;$$

where *D*_int_^low^ and *D*_int_^high^ are the distances between the centers of the adjacent pores in the AAO layer obtained at the low and high voltage, respectively. Taking into account that the experimentally found *D*_int_ values for the samples made using the voltage range from 20 to 80 V are well described as *D*_int_ = 15.4 + 2.2⋅*U*, the upper estimate for the fraction of the pores accessible for the electrodeposition in the templates was obtained (see solid line in Figure 3d). Thus, by varying the *U*_high_ one can obtain the AAO templates suitable for the formation of nanocomposites with the controllable metal volume fraction that is smaller than the template porosity. Our estimations of the minimum achievable volume fraction of metal in nanocomposites obtained by the suggested approach leads to the value of ca. 1% (if the first step of aluminum anodization is performed at 40 V in 0.3 M oxalic acid at 0 °C and then voltage increases to *U*_high_ = 140 V).

The experimentally observed fraction of the filled pores decreases from 80 to 35% when the anodization voltage increases from 40 to 60 V during the formation of the blocking layer. Note that the experimental values are smaller than the calculated ones (Figure 3d) even in the case of the constant anodization voltage. The difference between the theoretical and experimental values is related to the pores branching, which appears in the AAO film during the self-ordering process of the porous structure at the stage of potentiostatic anodization at 40 V [30]. For instance, in the s40 sample at least 59.4 ± 1.8% of the pores are straight and do not bifurcate during the growth of a 50 μm-thick AAO film under the chosen anodization conditions, whereas 20.3 ± 0.9% of the pores have terminated their growth due to branching of the same number of channels. For the s50 and s60 samples, the deviation of the experimental value from the calculated one is smaller than for the s40 sample, which is related to the smaller thickness of the AAO layer obtained at the constant voltage (20 μm for the s50 and s60 samples compared to 50 μm for the s40 sample). Consequently, during the formation of the first 20 μm of the film, 14.3 ± 1.9% of the channels are branched, and during the growth of the next 30 μm of the porous structure only 6.0 ± 2.8% of the pores are subjected to the bifurcation. The decrease in the rate of pore branching with the AAO film thickness agrees with the decrease in the rate of the porous structure self-ordering observed earlier [30]. It is worth noting that the analysis of the number of the filled channels from the side of the current collector is the direct method of the measurement of the fractions of straight, blocked, and branched channels, which is greatly important for the refinement of the theoretical models describing the processes of the templated electrodeposition [24,31] and different pressure-driven membrane processes where AAO films are implemented as membranes [20,32,33].

**Figure 4:**
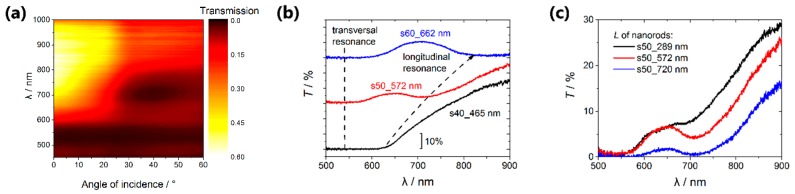
Angular-wavelength transmission spectrum of the sample s50_289 nm (**a**); transmission spectra of the samples with different volume fraction of metal (**b**) and with different nanorods lengths in the same template s50 (**c**). In all cases the incidence angle of 25° was used.

Optical properties of the hyperbolic metamaterials composed of an array of nanorods in a dielectric matrix were characterized by means of the transmission spectroscopy. The angular-wavelength transmission spectrum measured for the sample s50_289 nm is shown in Figure 4a. The two spectral features associated with the pronounced transmission minima can be distinguished here. The first one is the short-wavelength resonance centered at about λ = 530 nm, its quality factor is nearly independent on the angle of incidence. This spectral feature corresponds to the transversal localized surface plasmon resonance associated with the oscillations of the free electron gas in the direction perpendicular to the long axis of the nanorods [34]. The second (long-wavelength) plasmon resonance corresponding to the motion of the free electron gas along the metal nanorods was detected at the wavelength of about 710 nm. This longitudinal plasmon resonance can be observed only at the oblique incidence of the p-polarized radiation, which provides the existence of the electric field component of the pump field parallel to the long axis of the nanorods.

We found that optical properties of the composed hyperbolic metamaterials change significantly with the metal volume fraction as it is illustrated by Figure 4b, which shows the transmission spectra of the Au/AAO nanocomposites with a different volume fraction of the metal for the angle of incidence of 25°. The volume fraction of the metal in the s40_465 nm sample is so high (20%), that the short-wavelength and long-wavelength resonance modes cannot be resolved (Figure 4b, black curve). As the metal volume fraction decreases, the long-wavelength mode is controllably shifting to the red-wavelength range (from 600 to 900 nm), whereas the short-wavelength mode does not change its position (540 nm). This behavior of the system agrees with the theoretical treatment described below.

The other side of the coin is the disturbance of the hexagonal arrangement of the rods in the samples with partially blocked channels. This leads to an increase in the width of the resonance spectral bands. The width of the bands for the sample s40_465 nm is impossible to determine as the transverse and longitudinal resonances nearly coincide.

Using the s50 template, three samples with the average lengths of the Au nanorods *L* = 289 nm, 572 nm and 720 nm were synthesized, the corresponding spectra are shown in Figure 4c. It can be seen that the increase in the nanorods length in the specified range gives rise to the weak shift of the minimum position in the transmission spectrum of the nanocomposites, which corresponds to the longitudinal resonance from 690 to 720 nm, accompanied by a significant reduction of the transmission coefficient. Thus, the technique for the control of the fraction of the pores accessible for the electrodeposition proposed above is a more effective method of governing the optical properties of the hyperbolic metamaterials.

The spectra of the permittivity components of the composite metamaterials were calculated within the framework of the Bruggeman effective medium model for anisotropic media:

(2) $$\sum_{j=1}^{3}f_{j}\frac{\varepsilon_{i}^{eff}-\varepsilon_{j}}{\varepsilon_{i}^{eff}+L_{i}(\varepsilon_{i}^{eff}-\varepsilon_{j})}=0\;$$

where the subscripts i=∥,⊥ correspond to the directions parallel and perpendicular to the long axes of the nanorods, respectively; subscripts j=1, 2, 3 enumerate three components of the hyperbolic media under study, which are air, alumina and gold;$\;\varepsilon_{j}$ are the isotropic dielectric constants of these components taken from [35]; $f_{j}$ are the volume fractions of the components that were defined from SEM images of the samples; $L_{i}$ are the depolarization factors in the corresponding directions that can be calculated as $L_{∥}=\frac{1-e^{2}}{e^{2}}\left(\frac{1}{2e}\ln\left(\frac{1+e}{1-e}\right)-1\right)$, $L_{⊥}=\frac{1-L_{∥}}{2}$, $e^{2}=1-\frac{b^{2}}{a^{2}},\;\frac{b}{a}<1$ is a nanorods diameter-to-length ratio [34]. Equation (2) was numerically solved in the Wolfram Mathematica package for every wavelength in the range of 300–950 nm in order to obtain the spectra of the effective dielectric tensor components $\varepsilon_{⊥}^{eff}$ and $\varepsilon_{∥}^{eff}$.

**Figure 5:**
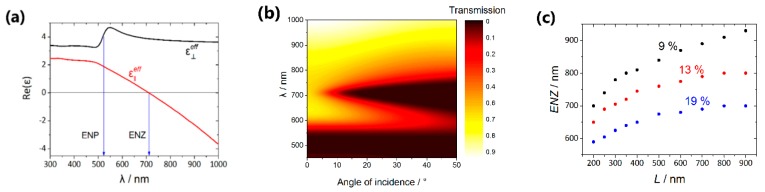
Calculation results. (**a**) Spectra of the permittivity components $\varepsilon_{⊥}^{eff}$ (black curve) and $\varepsilon_{∥}^{eff}$ (red curve) for the sample s50_289 nm with volume fractions of air, alumina, and metal of about 11%, 76%, and 13%, respectively. The nanorods length of 290 nm was taken; (**b**) angular-wavelength transmission spectrum for the sample s50_289 nm; (**c**) dependence of the ENZ spectral position on the length of the nanorods, the volume fraction of alumina is 76%, the volume fraction of gold is 9% (black points), 13% (red points) and 19% (blue points).

Figure 5a represents the calculated values of the permittivity components spectra for the nanocomposite s50_289 nm. Comparing these data with the transmission spectrum we can conclude that the transversal resonance corresponds to the pole of the $\varepsilon_{⊥}^{eff}$ component, whereas the transmission minimum associated with the longitudinal surface plasmon is observed in the spectral region, where $\varepsilon_{∥}^{eff}$ reverses its sign. Thus, the hyperbolic dispersion occurs for the wavelengths longer than the ENZ wavelength of approximately 710–720 nm.

Using the spectra of the permittivity components and Fresnel formulas the angular-wavelength transmission spectrum was calculated (Figure 5b). It is clear that the simulation results are qualitatively similar to the experimental transmission spectrum (Figure 4a). The long-wavelength resonance in the measured data is wider than that in the calculated ones due to the slightly different length of the nanorods in the samples under study.

The analogous calculations were performed for different volume fractions of gold and different values of the nanorods length, the corresponding results are presented in Figure 5c. Two items should be emphasized: (i) The ENZ position redshifts with the nanorods length increasing; (ii) the ENZ position shifts towards the shorter wavelengths with the increasing of the volume metal fraction due to the strengthening of the role of the interactions between the nanorods [34].

It should be also noted that these two tendencies are in good agreement with the experimental results (Figure 4b,c). The differences between the experimental and simulation data can be associated with the following reasons: (i) Imperfections of the effective medium approach used in the calculations, (ii) permittivity of the electrodeposited Au can differ from that of the bulk material that was taken for the simulations, (iii) inexact determination of the structural parameters from the SEM images.

## 4. Conclusions

The method for the synthesis of oriented arrays of Au nanorods in the dielectric matrix of anodic aluminum oxide with wide-range controllable metal volume fraction is proposed. The obtained materials, containing the oriented arrays of the nanorods with the diameter of 52 ± 5 nm and the length varying from 289 to 720 nm in dielectric matrix, exhibit the properties of hyperbolic metamaterials. Two bands of resonant light absorption corresponding to the excitation of the plasmon resonance transversely and longitudinally to the long axis of the nanorods are presented in the optical spectra of the samples. For the first time, the possibility to control the position of the longitudinal resonance in a wide range of wavelengths by the structural design of the template used for the nanorods electrodeposition is demonstrated. The increase in the anodization voltage leads to the formation of the blocked pores through the template thickness which remain empty during the metal electrodeposition in the case of the current collector coating on the upper surface of the oxide film. It is shown that when the metal volume fraction in the Au/AAO nanocomposite decreases, the redshift of the plasmon long-wave resonance occurs, and its position changes significantly stronger than in the case of the nanorods length variation.

From the methodological aspect, the statistical analysis of the filled and empty pores by the images of the nanocomposite surface after the selective removal of the current collector allows one to quantitatively determine the fraction of the straight, branched, and blocked channels in the AAO structure. This information is strongly needed for the refinement of the existing model descriptions of the AAO structure which are used for the building of the theoretical models of template electrodeposition as well as of different pressure-driven membrane processes in the AAO channels.

Experimental results were supported with calculations of the permittivity tensor components within the effective medium approximation. The calculated spectra proved the existence of the ENZ point at 710–720 nm, so that the hyperbolic regime for the structures under study corresponds to longer wavelengths. The model calculation also confirms that the ENZ spectral position redshifts as the nanorods length increases; whereas ENZ shifts in the opposite spectral range for the composite metamaterial with a larger volume metal fraction.

The applied synthetic approach makes it possible to obtain nanocomposites with the metal volume fraction smaller than the porosity of the matrix. The realistic value of the minimum volume fraction of metal in nanocomposites based on AAO templates prepared in 0.3 M oxalic acid electrolyte is 1%. Nanocomposites with a small content of an inclusion phase are prospective for the optical and catalytic implementations.
